# The architecture of chicken chromosome territories changes during differentiation

**DOI:** 10.1186/1471-2121-5-44

**Published:** 2004-11-22

**Authors:** Sonja Stadler, Verena Schnapp, Robert Mayer, Stefan Stein, Christoph Cremer, Constanze Bonifer, Thomas Cremer, Steffen Dietzel

**Affiliations:** 1 Department Biologie II, Biozentrum der Ludwig-Maximilians-Universität München, GroßhadernerStraße 2, 82152 Planegg-Martinsried, Germany; 2 Kirchhoff Institut für Physik, Universität Heidelberg, 69120 Heidelberg, Germany; 3 Molecular Haemopoiesis and Epigenetics Group, St. James's University Hospital, University of Leeds, Leeds LS9 7TF, UK

## Abstract

**Background:**

Between cell divisions the chromatin fiber of each chromosome is restricted to a subvolume of the interphase cell nucleus called chromosome territory. The internal organization of these chromosome territories is still largely unknown.

**Results:**

We compared the large-scale chromatin structure of chromosome territories between several hematopoietic chicken cell types at various differentiation stages. Chromosome territories were labeled by fluorescence in situ hybridization in structurally preserved nuclei, recorded by confocal microscopy and evaluated visually and by quantitative image analysis. Chromosome territories in multipotent myeloid precursor cells appeared homogeneously stained and compact. The inactive lysozyme gene as well as the centromere of the lysozyme gene harboring chromosome located to the interior of the chromosome territory. In further differentiated cell types such as myeloblasts, macrophages and erythroblasts chromosome territories appeared increasingly diffuse, disaggregating to separable substructures. The lysozyme gene, which is gradually activated during the differentiation to activated macrophages, as well as the centromere were relocated increasingly to more external positions.

**Conclusions:**

Our results reveal a cell type specific constitution of chromosome territories. The data suggest that a repositioning of chromosomal loci during differentiation may be a consequence of general changes in chromosome territory morphology, not necessarily related to transcriptional changes.

## Background

It is a longstanding observation that chromatin distribution in the interphase cell nucleus varies with the cell type. Flemming described differences in nuclear appearance in 1882 [[1], p.100]. Since then methodological advancements have made it possible to study nuclear chromatin architecture in much more detail. The spatial restriction of each chromosome to a limited area of the interphase nucleus, the chromosome territory, has been unequivocally demonstrated by fluorescence in situ hybridization (FISH) [2,3]. However, although progress has been made over the last decade [for reviews see [4-7]], the internal organization of chromosome territories is still largely unknown. Here we asked whether chromosome territories display differences between cell types in their internal chromatin organization.

We amended our experimental approach with the determination of the position of a gene locus relative to its chromosome territory. In several previous studies it was observed that a number of active genes located preferentially at the surface of their chromosome territories or even outside [8-12], while others noted that active genes could also be positioned in the chromosome territory interior [13]. A general labeling of transcription sites resulted in signals throughout chromosome territories [14,15] demonstrating that the periphery of chromosome territories is not the only region where transcription occurs. Fluorescence in situ hybridization (FISH) studies investigating the major histocompatibility complex MHC [10] or the epidermal differentiation complex EDC [11] showed looping beyond the surface of the chromosome territory upon activation in up to 25% of the cases. Both loci are in the megabase size range with multiple co-regulated genes. Even higher frequencies of a location outside the territory were described for a gene rich human region without coordinate gene expression on chromosome 11p15.5 [16] and for genes of the Hoxb cluster in mouse embryonic stem cells entering differentiation [12]. It has thus been suggested that strongly expressed genes may be on chromatin loops that loop to the periphery of the territory, while genes expressed at low levels may occupy either a more interior or a random position [13]. Difficult to interpret were data concerning looping out of the β-globin gene locus from its chromosome territory in mouse erythroleukemia cells. In unstimulated cells where the locus shows DNase-hypersensitive sites but is not yet expressed, nearly half of the loci looped out. In stimulated cells where expression occurs, however, this was found only in about a third of the cases [17]. Consistent with the looping out of endogenous loci found in FISH studies, an opening of GFP-labeled artificial chromosomal regions was observed upon transcriptional activation or binding of transcription factors [18-24].

**Figure 1:**
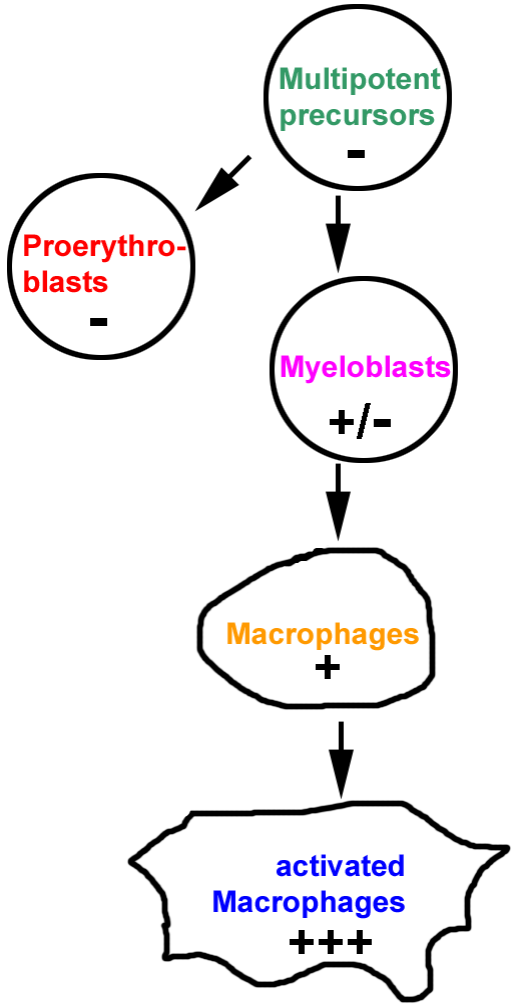
Cell lines used in this study. Pluses and minuses indicate the expression state of the lysozyme gene. Colors are the same as used in Figures 4, 5 and 7.

So far, a correlation of gene activation with increasing looping-out from the chromosome territory has only been shown for gene clusters but not for single genes. In the present study we have chosen the chicken lysozyme gene (*cLys*), which is highly active in macrophages, as a model system to explore the possibility of positional changes during activation of a single gene. *cLys *does not have co-regulated neighbors. Recently the gene *cGas41 *was found only 200 bp downstream of the polyA-site of *cLys*. *cGas41 *is expressed on a low level in all chicken tissues and cell lines tested, including all cell lines used here [25]. Macrophage differentiation is an interesting model system for studies of cell fate decisions. As all blood cells, macrophages originate from pluripotent hematopoietic stem cells and develop via defined multipotent and then progressively restricted precursor types. The developmental regulation of lysozyme in this differentiation system is well characterized. Expression is not detectable in multipotent myeloid precursors, which are able to differentiate to either the erythroid, granulocytic or the macrophage lineage (Figure 1). The gene is also not expressed in the erythroid lineage. Expression is first detected at a low level in granulocyte-macrophage precursors (myeloblasts) and is further upregulated in macrophages. By the addition of bacterial lipopolysaccharide (LPS) to macrophages, another tenfold increase in lysozyme expression is caused [26]. Studies of *cLys *regulation were greatly facilitated by cell lines representing these differentiation stages [27,28].

The chicken karyotype consists of several pairs of so called macrochromosomes with sizes comparable to that of mammalian chromosomes and many much smaller microchromosomes [for review see [29,30]]. *cLys *is located on the short arm of chromosome 1 which is with about 190 Mbp comparable in length to human chromosome 4 [31].

In the present study, we investigated the large-scale chromatin organization of chromosome territories in well characterized cell lines representing the five chicken cell types described above (Figure 1). Multi-color 3D FISH was applied to cells with structurally preserved nuclei, followed by confocal microscopy and three-dimensional image analysis. We assessed the morphology of chromosome territories 1 and 8 by visual inspection and measured the dispersion of the painted territories in each cell type. We demonstrate that chromosome territory dispersal increases in the more differentiated cell types. Further, we determined the 3D positions of the chicken lysozyme gene domain (including *cGas41*) and the chromosome 1 centromere relative to their chromosome 1 territory. We found that not only the lysozyme gene domains but also the centromeres were mostly in the chromosome territory interior in multipotent myeloid precursor cells and relocated to the territory periphery in further differentiated cell types. In addition, we determined the radial positioning of the chromosome territories 1 and 8 within the nuclei of each cell type and measured nuclear volumes.

## Results

### The morphology of chromosome territories changes during differentiation

**Figure 2:**
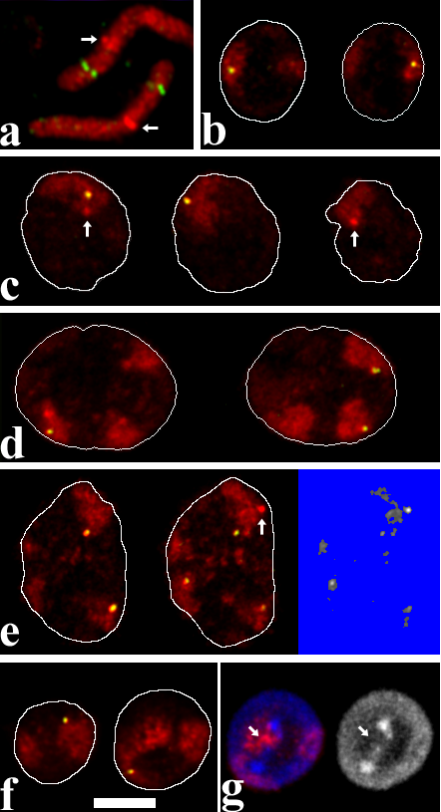
FISH with a chromosome 1 paint probe (red) and the lysozyme gene domain probe (green/yellow). (a) FISH on metaphase chromosomes. The chicken lysozyme gene domain is located on the short arm of chromosome 1. Note that the library probe mix used gives particularly strong signals at the centromeres (arrows). (b-g) 3D-FISH on structurally preserved nuclei. For each cell type, single confocal sections of one nucleus are shown. In b-f, nuclear outlines were drawn after the DNA counterstain which was omitted from the figure to avoid obstruction of the territory signals. In addition to the *cLys *domain signals, centromeres are in focus in some of the sections (arrows). (b) Multipotent myeloid precursor cell. (c) Myeloblast. (d) Macrophage without LPS-activation. (e) Macrophage with LPS-activation. On the right hand side, a threshold of 80 was applied to the territory signal of the central image to visualize disaggregation into several objects. While usually only few objects are present in any given focal plane, in this particular example the breakup is well recognizable. The algorithm applied in the calculations works on 3D-stacks, however. The macrophage cell line is aneuploid (see Methods), the cells shown in d and e have three territories with chromosome 1 material, each containing a *cLys *signal. (f) Erythroblast. (g) An additional section of the erythroblast shown in f visualizes a cluster of chromosome 1 material (red in left image) in a central nuclear area (arrow) with low DNA-counterstain but associated with a brightly stained region (see main text). Such clusters were less pronounced when only paint probes from early DOP-PCR-amplification rounds were used (see Methods for details). DNA counterstain is blue in left, gray in right image. Scalebar 5 μm for b-g. Whereas in precursor cells the lysozyme gene domain signal was found nearly always inside the territory, in differentiated cells more external positions were frequent. Note that the multipotent myeloid precursor cell has a relatively small nucleus and nuclear volume is increased in the further differentiated cell types.

**Figure 3:**
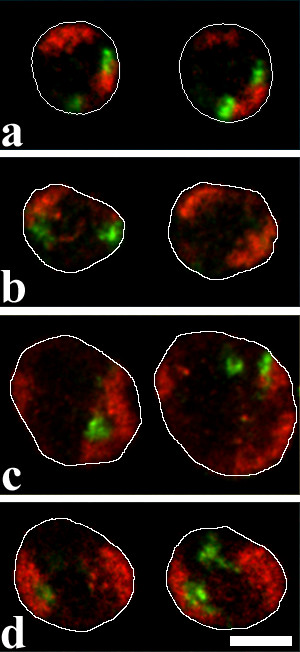
3D-FISH on structurally preserved cell nuclei with paints for chromosome 1 (red) and chromosome 8 (green). For each cell type, two confocal sections of one nucleus are shown. Nuclear outlines were drawn after the DNA counterstain which was omitted to avoid obstruction of the territory signals. (a) Multipotent myeloid precursor. (b) Myeloblast. (c) Macrophage activated with LPS. (d) Proerythroblast. Scalebar 5 μm.

**Figure 4:**
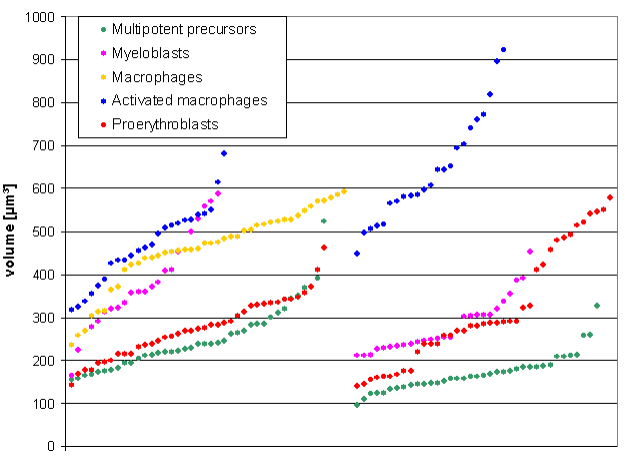
Nuclear volumes. Each dot indicates the volume of one nucleus. Nuclei from experiments with hybridization of chromosome 1 and *cLys *probes (left) and those with chromosome 1 and 8 probes (right) were sorted by size to allow an easier comparison by eye. See main text for mean values.

3D FISH was performed on formaldehyde fixed, structurally preserved nuclei. Visual examination of painted chromosome 1 and chromosome 8 territories revealed differences between the cell types (Figure 2, Figure 3). Territories in multipotent myeloid precursor cells were relatively compact and homogeneously stained (Figure 2b, Figure 3a). In proerythroblasts, territories were more diffuse and borders became less definable (Figure 2f,2g, Figure 3d). These changes cannot be explained with an increase in nuclear volume since nuclei of proerythroblasts were smaller than those of myeloblasts (see below and Figure 4). In a minority of proerythroblasts, in addition to the labeled territories our paint probe labeled DNA-clusters in the center of the nucleus. These clusters had low DNA counterstain but were often associated with strongly counterstained regions (see arrow in Figure 2g). The clusters may play a role in forming heterochromatin as observed during differentiation of human erythroid cells [4]. Chromosome territories in myeloblasts (Figure 2c, Figure 3b) appeared less compact than in precursors but more compact than in proerythroblasts. Territories in unstimulated macrophages (Figure 2d) had even more diffuse borders. In their interior we observed agglomerations of labeled DNA and lacunas in some cases. A maximum of dispersal was noted in stimulated macrophages (Figure 2e, Figure 3d). Here, territories had grooved, fuzzy surfaces and a heterogeneous label throughout. Lacunas were frequent in the larger chromosome 1 territories.

**Figure 5:**
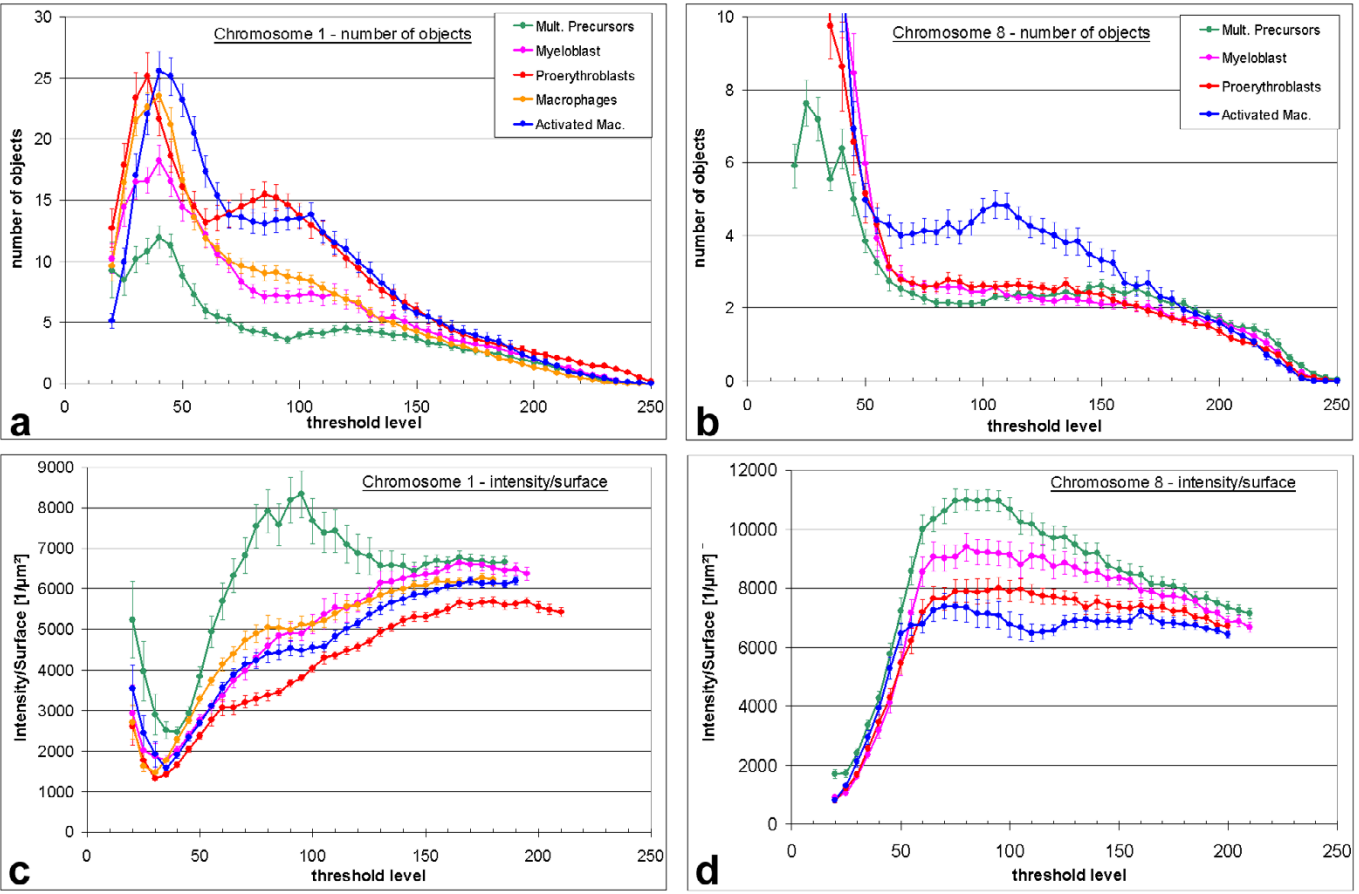
Disaggregation of chromosome territories in objects. (a,b) mean number of objects at increasing thresholds for chicken chromosome 1 (a) and chromosome 8 (b) territories. When the starting threshold of 20 is gradually increased, the nuclear background produces at first few and then many objects (around threshold 40). Suppression of nuclear background occurs at thresholds between 60–70, leaving chromosome territories only. The range above these thresholds is thus the most interesting since it is here where the territories start to break up in several objects (compare Fig. 2). These objects are gradually lost at further increasing thresholds. Values for macrophages are not directly comparable to other cell types since they contain additional chromosomes (see Methods). (c, d) Chromatin content per surface. Signal intensity of objects was divided by object surface area and averaged (see Methods for details). Since additional chromosomal parts add intensity as well as surface, this parameter is unsusceptible to aneuploidy.

A comparison of chromosome territory surface and chromatin texture by visual inspection is bound to subjective influences. To allow an unbiased, quantitative evaluation of chromosome territory morphology, we counted the number of objects to which chromosome territories disaggregate at increasing threshold levels. With a computer program newly developed for this purpose (for details see Methods and Figure 2e) we could confirm the visual impression of relatively compact chromosome 1 territories in multipotent precursors by showing that at higher thresholds they disaggregate to smaller numbers of objects than the fuzzier territories of more differentiated proerythroblasts (Figure 5a). Statistical inferences about the means of the maximal number of objects in these three cell types were highly significant as determined by Analysis of Variance (one-way ANOVA; F(2,119) = 58.5, p < 0.001; see Methods for details). Post-hoc Sidak tests revealed a significant difference between precursor cells and myeloblasts (p = 0.013) and highly significant differences between proerythroblasts and the two other cell types (p < 0.001). Chromosome 1 territories in macrophages also yielded high numbers of objects. However, macrophages of the utilized cell line contain an additional fragment of the short arm of chromosome 1 and sometimes complete additional chromosomes 1. Due to this aneuploidy object numbers are biased towards higher numbers. Results are thus not directly comparable with other cell lines. Notably, in stimulated macrophages territories disaggregate into more objects than in unstimulated ones, suggesting that stimulation triggered a change to a more dispersed chromatin texture (p < 0.001). The stronger dispersion of chromosome 1 territories in more differentiated cells was confirmed in a second experimental series with painted chromosome 1 and chromosome 8 territories (Figure 3) in multipotent precursors, proerythroblasts, myeloblasts and activated macrophages (data not shown). The DNA content of chicken chromosome 8 is about 30 Mbp [31]. This is roughly one sixth of the DNA content of chicken chromosome 1 and about two thirds of the smallest human chromosome, 21. Chromosome 8 was diploid in all utilized cell lines. As expected due to its smaller size, it disaggregated in all cell types to a much smaller number of objects (Figure 5b). ANOVA analysis of all groups showed a highly significant aberration from the assumption of similar distributions in all cell types (F(3,116) = 38.2, p < 0.001). A difference between multipotent precursors, proerythroblasts or myeloblasts was not detectable (p > 0.9 in post-hoc Sidak tests) but in activated macrophages chromosome 8 territories did break up to a larger number of objects than in other cell types over a wide threshold range (p < 0.001 with all other cell lines).

To allow a comparison of chromosome 1 territories in the aneuploid macrophages with those of other cell types, we analyzed the intensity of objects, i.e. their chromatin content, per surface area (Figure 5c,5d, see Figure legend and Methods for details). The chromatin content per object surface area was measured in multipotent precursor cells for both, chromosomes 1 and 8, again confirming their more compact structure in this cell type as compared to more differentiated cells. As a third parameter, we measured the average amount of labeled chromatin (signal intensity) per voxel (volume pixel) of the segmented objects. This parameter did not show differences between the cell types. Thus in all cell types a given amount of chromatin within the segmented objects was distributed over a similar volume over a wide threshold range (data not shown).

### The positions of the lysozyme gene domain and of the chromosome 1 centromere change during myeloid differentiation

To determine the positioning of the lysozyme gene domain relative to the chromosome 1 territory, we performed dual color FISH with a chromosome 1 paint probe and a 20 kb plasmid probe for the lysozyme gene domain (Figure 2). By using a particular probe mix (see Methods) we were able to obtain an especially bright signal at the centromere in the same color channel as the paint probe.

**Figure 6:**
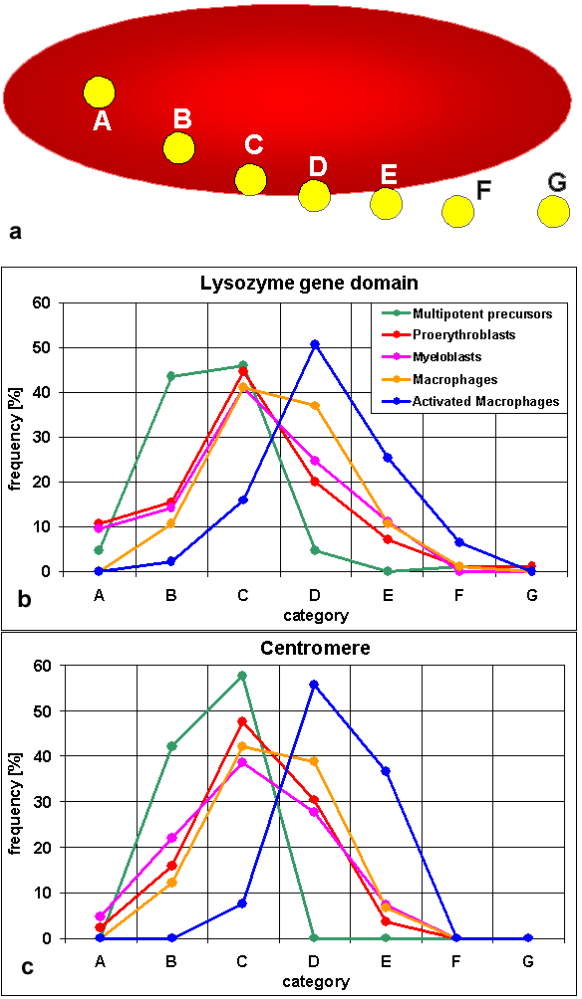
Classification of *cLys *gene domain and centromere signals. (a) Scheme used to classify the localization of gene and centromere signals relative to their chromosome 1 territory [adopted from 11]. The red ellipsoid represents the territory, the yellow dots the signals of genes or centromeres. Categories are: A, inside the territory delineated by the paint probe, away from the surface. B, inside, closer to the territory surface but not touching it. C, inside and touching the surface. D, on the surface. E, outside and touching the surface. F, without contact to the territory but in immediate neighborhood. G, away from the territory. (b, c) Distribution of the lysozyme gene domain (b) and the centromere (c) relative to the chromosome 1 territory in 5 different cell types. Between 79 and 95 *cLys *gene domain and centromere signals were evaluated for each cell line and assigned to the categories A-G.

The positions of both, the lysozyme gene domain and the centromere, differed largely between the cell lines representing the various differentiation stages. To classify the positions of the signals, we used the scheme shown in Figure 6a. In multipotent precursor cells (Figure 2b) we found the *cLys *gene domain inside the harboring chromosome territory, away from the territory border (categories A and B) in 48% of the cases (Figure 6b). In additional 46% the gene signal was inside the territory touching the border (cat. C). It was previously shown that these cells do not express lysozyme but do show low level expression of the neighboring *cGas41 *[25]. We conclude that a location inside the territory is compatible with low-level expression. In further differentiated cells, the lysozyme gene locus was found more often in the periphery of chromosome 1 territories (Figure 2, Figure 6b). This is true for myeloblast/macrophage lineage cells with lysozyme expression as well as for proerythroblasts in which the expression of *cLys *and *cGas41 *does not differ from the precursor cells. The most peripheral localization, sometimes outside of the painted territory, was found in activated macrophages (Figure 2e) where the *cLys *expression level is highest. Here 82% of the gene signals were on the surface or further outside (cat. D-F). The difference in distribution between precursor cells and all other cell types was highly significant (p < 0.001) as was the difference between activated macrophages and all other cell types (p < 0.001). The difference between unstimulated macrophages and proerythroblasts (p = 0.003) or myeloblasts (p = 0.024) was also significant whereas the difference between myeloblasts and proerythropblasts was not (p = 0.5). To test the robustness of our results, we repeated statistical analysis after reducing the number of applied categories of localization to only three: internal (A+B), peripheral (C-E) and external (F+G). We confirmed highly significant differences when precursor cells or activated macrophages were compared to any other cell type (p = 0.003 or smaller). In summary, for the lysozyme gene we found a change in position from interior when not expressed in myeloid precursor cells to peripheral when strongly expressed in activated macrophages. This would fit the hypothesis that highly expressed genes are preferentially located in the territory periphery, as it was found previously for large gene clusters [10,11]. However, this hypothesis does not explain the difference in positioning between the precursors and the proerythroblasts.

Surprisingly, the centromeres of chromosome 1 showed a change in distribution very similar to the *cLys *gene domain (Figure 6c). In no cell type we found a significant difference between the two (p = 0.255 or larger). For example, in multipotent precursors all detected centromeres were inside the territory, either without (cat. A, B) or with contact to the surface (cat. C). In contrast, in activated macrophages 93% of the centromeres were on the surface (cat. D) or outside with contact to the surface. Again, the difference in distribution between precursor cells and all other cell types was highly significant (p < 0.001) as was the difference between activated macrophages and all other cell types (p < 0.001). Myeloblasts and unstimulated macrophages showed a moderately significant difference (p = 0.044) whereas the differences between proerythroblasts and myeloblasts (p = 0.654) or unstimulated macrophages (p = 0.084) were not significant. When applying only three categories of localization as described above, differences between precursor cells or activated macrophages and any other cell type again where highly significant (p = 0.001 or smaller) with the exception of precursor cells compared to myeloblasts showing a moderate significant difference (p = 0.035).

### Silent lysozyme genes do not colocalize with centromeric heterochromatin

Brown et al. [32,33] showed examples of genes in hematopoietic cell types, which were tethered to centromeric heterochromatin when silent, but located remote from heterochromatin when active [4,32,34]. We asked whether the same nuclear location could be found for silent and active lysozyme genes. A probe that would label all centromeres of chicken chromosomes is not available. We reasoned that if the inactive lysozyme gene would be tethered to centromeric heterochromatin, at least in a number of cases this centromeric heterochromatin should include the centromere of its own chromosome. High precision 3D-distance measurements [35,36] from the lysozyme gene domain to the corresponding chromosome 1 centromere in the data sets described above showed that there is no such colocalization (Table 1). In those cell types where the lysozyme gene is completely shut off, the smallest distances found were 0.6 μm in proerythroblasts and 0.5 μm in multipotent precursor cells. This finding rules out a colocalization of the two loci. Distances in multipotent precursors are on average somewhat smaller than in the other cell types (Table 1). This can be attributed to a more compact chromosomal shape and to a smaller nuclear volume in this cell type (see below).

**Table 1:** 3D-Distance measurements between the lysozyme gene domain and the centromere of the corresponding chromosome 1 in interphase nuclei of different cell lines.

	**multipotent precursor cells**	**proerythroblasts**	**myeloblasts**	**macrophages**	**LPS induced macrophages**
evaluated territories	70	85	80	89	78
mean value	1,5 μm	2,1 μm	2,5 μm	2,2 μm	2,2 μm
median	1,4 μm	2,0 μm	2,2 μm	2,2 μm	2,1 μm
Standard-deviation	0,8	0,8	1,1	0,8	0,9
Smallest value	0,5 μm	0,6 μm	0,7 μm	0,7 μm	0,5 μm
Largest value	4,8 μm	5,8 μm	5,3 μm	4,4 μm	5,1 μm

### Radial positioning of chromosome territories 1 and 8 within the nucleus

**Figure 7:**
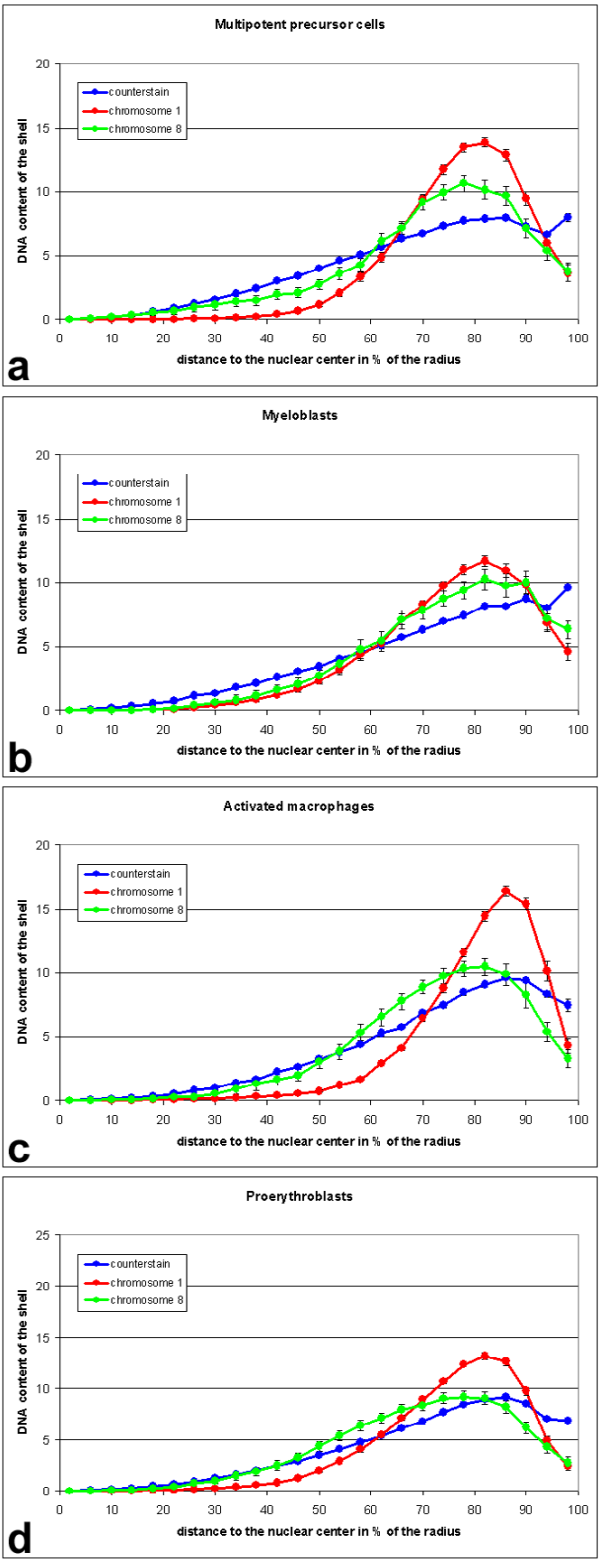
Radial distribution of chromosomes 1 (red) and 8 (green) in nuclei of (a) multipotent precursor cells (n = 37), (b) myeloblasts (n = 27), (c) activated macrophages (n = 23) and (d) proerythroblasts (n = 40). Unlike in the median distribution used for determination of significance levels, in the graphs shown here all voxels of a segmented signal are represented. Chromosome 8 has only about one sixth of the size of chromosome 1. Accordingly, its interphase territories are much smaller, leading to a smaller sample of voxels and thus accounting for less smooth curves than for chromosome 1 territories, e.g. in myeloblasts. All curves for each chromosome are shown in one graph in a supplemental figure in additional file 1.

Habermann et al. [37] showed that in embryonic chicken neuronal and fibroblast nuclei the gene poor macrochromosomes 1–5 are located at the nuclear periphery. Intermediate chromosomes 6–10 were found further inside but not as central as the gene rich microchromosomes. Respective results were also found in human and other primate cells [38-43]. According to the current release of the chicken genome sequence [31] chromosome 1 has a length of 188 Mbp with ~11 genes/Mbp and chromosome 8 has 30 Mbp with ~19 genes/Mbp. These numbers are likely to change somewhat with further releases of the sequence. They do suggest however that the relative gene content is higher for the smaller chromosome 8. To test for a difference in the radial distribution of individual chicken chromosome territories, we measured 3D radial distributions in the nuclei painted with chromosomes 1 and 8 from experiments described above (Figure 7).

In multipotent myeloid precursor cell nuclei, chromosome 1 territories were located more peripheral than chromosome 8 territories (p < 0.005). The same was true for proerythroblasts (p < 0.001) but no significant difference was present in myeloblasts (p > 0.1). These three cell types grow in suspension and have round nuclei. In flat nuclei of LPS-stimulated macrophages we again found chromosome 1 territories more peripheral than chromosome 8 territories (p < 0.005). The radial distribution is also reflected by the signal median values. It indicates at which nuclear radius half of the signal voxels are more internal and half are more external. In 73% of the precursor cells the chromosome 1 signal median is more external than the chromosome 8 signal median. The respective values are 52% for myeloblasts, 82% for proerythroblasts and 91% for activated macrophages.

When comparisons between cell types were made, chromosome territories 8 showed a rather similar radial distribution in all cell types (p > 0.1 or >0.05 for all combinations with a frequency maximum of ~10% at or near 80% of the nuclear radius, Figure 7, supplemental figure in additional file 1). Chromosome 1 territory distribution did show significant differences between cell types (Figure 7, supplemental figure). Chromosome 1 territory radial distribution was compared between nuclei co-hybridized either with the *cLys*-probe (series 1, Figure 2, not shown as graph) or the chromosome 8 paint probe (series 2, Figure 3 and Figure 7). In LPS-stimulated macrophages chromosome 1 territories were further outside than in proerythroblasts (p < 0.001 in series 1 and series 2), in myeloblasts (p < 0.001 in series 1 and p < 0.01 in series 2) and in precursors (p < 0.01 in series 1 and p < 0.05 in series 2). No significant difference was found between stimulated and unstimulated macrophages (p > 0.1, series 1 only). A significant difference in radial distribution of chromosome 1 territories between precursors and myeloblasts was found in series 1 (p < 0.01) but not in series 2 (p > 0.1). The same was true for the comparison of precursors and proerythroblasts (series 1: p < 0.001; series 2: p > 0.1). Proerythroblasts and myeloblasts did not show a significant difference (p > 0.1 in both series).

The radial distribution of the lysozyme gene domain did not differ significantly between the cell lines (p > 0.1). The mean value of the signal medians was between 71 and 76% of the nuclear radius for all cell lines. In erythroblasts, chromosome 1 territory signal medians were more internal than *cLys *signal medians (66% vs. 72%, p < 0.005). In unstimulated macrophages the opposite was true (76% vs. 73%, p < 0.05). In the other cell types the differences between the signal medians of *cLys *and chromosome 1 territories were between 0–2% and not significant.

Our results are compatible with previous data [37] in describing an external location for chromosome 1 and a somewhat more internal location for chromosome 8. In addition we find differences in the radial distribution of chromosome territories between the chicken cell types that have not been observed previously.

### Nuclear volumes

The volume of nuclei was measured in confocal stacks of DNA counterstain of data sets described above by the same program that was used for the calculation of the radial distributions (Figure 4). The mean value for nuclear volumes for multipotent myeloid precursors was 212 μm^3 ^(± 75 standard deviation, n = 76) and for myeloblasts 327 ± 101 μm^3 ^(n = 50). The difference between the two cell types was highly significant (p < 0.001). The mean nuclear volume of proerythroblast was 296 ± 109 μm^3 ^(n = 78). Proerythroblasts did not consistently show significant differences when compared to precursor cells or myeloblasts. Since the macrophage cell line carries additional chromosomes, its nuclear volume cannot be directly compared to the other cell lines. For unstimulated macrophages we determined nuclear volumes of 459 ± 91 μm^3 ^(n = 42) and for LPS stimulated macrophages 554 ± 139 μm^3 ^(n = 78). This difference was not significant.

The measured nuclear volume depends on the chosen signal threshold. Since the volume increases by the power of 3 with the nuclear radius, small differences in the segmentation can lead to large volume differences. A cautious interpretation of such measurements is thus advised. The difference between multipotent myeloid precursors and myeloblasts is so large however that we are confident that it is real and not a thresholding artifact.

## Discussion

Cell type specific chromatin distributions on the nuclear level have been described for over a century [[1], p.100]. Differences between cell types have also been described for the distribution of heterochromatin detected with antibodies against methylated histones [44], for the radial distribution of gene rich and gene poor chromosomes [[37,40,45] this study] and the occurrence of clustering between specific chromosome territories [45]. Here we show an example were large-scale chromatin organization of chromosome territories changes during differentiation, and thus add a new feature to the list of nuclear architectural properties that can differ between cell types.

To quantify chromatin dispersal of labeled chromosomes in cells of various differentiation stages, we counted the number of separate, labeled chromatin objects to which the chromosome territories disintegrated at increasing thresholds. In the investigated chicken cell types, chromosome territories of further differentiated cell types disaggregated into more objects. An increase in object number during differentiation may indicate that a significant number of compact chromatin domains with silent genes separate from each other into several, more decondensed, "open" chromatin domains. This would increase the accessibility to transcription factor complexes from the interchromatin compartment by increasing the available chromatin surface area of the chromosome territory. In human lymphoblasts gene rich chromosome 19 territories were found more decondensed than gene poor chromosome 18 territories [38] and electron microscopic evidence suggests that active genes are exposed at chromatin domain surfaces in a zone called the perichromatin region, a transitional zone that marks the transition form the chromatin domain periphery to the interchromatin compartment [46]. A caveat of this interpretation is that so far no unequivocal proof for a profound influence of higher order chromatin compaction on gene activation and gene silencing has been presented. A further possibility is that inactive loci in the more differentiated cells do not require a tight spatial silencing by chromatin compaction anymore because the set of available molecular activators and repressors has changed. At present we can only speculate whether the correlation of increased dispersal of chromosome territories with differentiation state is a widespread feature or restricted to a few chicken blood cell types. At the highest, nuclear level of chromatin organization it was described for mammalian nuclei that heterochromatin shows distinct patterns of large blocks in terminally differentiated cells but not in blood stem cells and tumor cells [47,48]. This indicates a compaction of chromatin in differentiated cells rather than in their precursors, unlike in our current data on the chromosome territory level. It is possible, that heterochromatin (consisting mainly of repetitive sequences) and the bulk of labeled chromosome territories behave differently in these aspects. Due to their suppression with unlabeled repetitive DNA, repetitive sequences are underrepresented in chromosome territories detected by FISH as in the present study. Also, the rather small amount of repetitive sequences and heterochromatin in the chicken genome (genome size ~1.2 Gbp according to [29], 1.1 Gbp according to [31]) as compared to mouse and human genomes (~3.2 Gbp each, [31]) may lead to differences in nuclear organization.

Multipotent myeloid precursor cells have the smallest nuclei of the cell types investigated here. Myeloblasts have on average larger nuclei than proerythroblasts. If the observed disaggregation of chromosome territories were based on a nuclear volume increase, the larger myeloblast nuclei should have a stronger dispersion of chromosome territories than proerythroblasts. However, the opposite is true (Figure 5). We thus conclude that chromosomal dispersion is not related to nuclear size. In general, we observed larger nuclear volumes for further differentiated cell types. Increasing nuclear size was also observed during maturation of nerve ganglia cells [49] while a volume decrease was described during the maturation of lymphocytes [47]. Accordingly, unlike recently suggested [50], a decrease of nuclear size does not appear to be a phenomenon generally associated with terminal differentiation events.

The lysozyme gene domain is positioned inside the chromosome 1 territory in multipotent myeloid precursor cells where the lysozyme gene is inactive, but on the surface or outside in most of the territories in activated macrophages where the gene is strongly expressed. We thus did find a tendency to more exterior regions of the chromosome territory for the highly expressed gene from in activated macrophages although actual looping-out (without visible contact to the territory) was observed in only about 6%. Interestingly, while the radial distribution of the lysozyme gene domain within the nucleus is about the same in all cell types, the harboring chromosome 1 territories show differences. The finding that in erythroblasts the *cLys *signal is more exterior than the chromosome 1 territory signal median but in unstimulated macrophages the opposite is true also argues for a cell type specific organization of chromosome territories. A similar observation has been described for a IL-3 induced differentiation of human leukemic K562 cells where the β-globin gene cluster does not change nuclear position but the harboring chromosome 11 territory does [51]. However, in human hematopoietic cells a relocation of a gene to a different preferential radial position [52] or to or away from heterochromatic nuclear compartments has been observed for some genes, correlated with transcriptional regulation at different developmental stages [e.g. [33,53]]. Unfortunately, the harboring chromosome territories were not labeled in these studies.

While we can exclude a tethering of the inactive lysozyme gene to the centromere, at first glance this result seems compatible with the hypothesis that inactive genes are stored away in internal regions of the chromosome territory and active genes are on their surface or even looped out. However, several aspects suggest an alternative explanation. (i) Embedded in the chicken lysozyme gene domain is a second gene, *cGas41*, which, albeit on a low level, is expressed in all cell types used in this study, including multipotent myeloid precursor cells [28]. Thus we found an example of an active gene with a location inside the territory as it was described previously for some mammalian genes [13]. (ii) Although the position of the lysozyme gene domain is most peripheral in activated macrophages where the expression is highest, we also found a shift towards more external positions from multipotent myeloid precursor cells to further differentiated proerythroblasts, both non-expressing cell types. (iii) In addition to the lysozyme gene domain, we investigated the chromosome 1 centromere. Surprisingly, both loci showed a very similar distribution in all cell types investigated. Transcription from centromeres has been observed in yeast [reviewed in [54]] and from a human neocentromere [55]. Formally, we thus cannot fully exclude that centromeric transcription may occur in chicken. We regard it as extremely unlikely, however, that tissue dependent differences in centromeric transcription play a role in the cell type specific spatial positioning found here. The observed modification in the morphology of chromosome territories during differentiation rather invites to hypothesize that the positional changes observed for the lysozyme gene domain are not restricted to this particular chromatin loop or only to those chromatin loops which harbor genes that become activated during cell differentiation. Instead, these positional changes may reflect a more general, differentiation dependent change in large-scale chromatin structure. Differentiation processes may thus have a more global impact on chromatin structure than previously suspected.

## Conclusions

We describe several features of chromosome territory organization that differ between various hematopoietic chicken cell lines. While multipotent myeloid precursor cells had compact chromosome territories, the more differentiated cell types investigated here displayed somewhat disaggregated, diffuse territories. Although nuclear volumes generally are larger in the more differentiated cell types, they do not correlate with the changes in chromosome territory morphology. The chicken lysozyme locus as well as the chromosome 1 centromere is located preferentially in the interior of the chromosome territory in precursor cells and more external in more differentiated cells. Our data suggest that such a repositioning of chromosomal loci during differentiation may be a consequence of general changes in chromosome territory morphology, not necessarily related to transcriptional changes. The radial distribution of chromosomes 1 and 8 also differed between cell types. In summary our data argue for a cell type specific chromosome territory organization in the investigated cell lines.

## Methods

### Cells

All cells are from retrovirally transformed chicken cell lines [56,57]. HD50MEPs represent multipotent myeloid precursor cells before the separation of erythroid and monoblast/macrophage lineages. Proerythroblast-like HD37 were used as an example for a differentiated cell type that is negative for lysozyme expression. Myeloblasts (HD50 myl, a cell line derived from the same precursors as HD50MEP) are an intermediate stage in the differentiation to macrophages (HD11). While macrophages grow adherent, all others grow in suspension. Cytogenetic analysis showed that all lines were diploid for chromosome 1 except HD11. The latter had 2 to 4 normal chromosomes 1 plus a translocation chromosome with the short arm of #1 including *cLys *but not the #1 centromere and also other karyotype aberrations, e.g. a trisomy of chromosome 2. All lines were diploid for chromosome 8. Cells were cultured in IMDM supplemented with 2% chicken serum, 8% fetal calf serum and 2 mmol L-Glutamin and maintained at 37°C with 5% CO_2_. For 3D-FISH experiments cells were cultured on #1.5 glass coverslips (170 μm thick). Except for macrophages, coverslips were pretreated for 35 min with poly-L-lysine (0.1 mg/ml; MW 300000, Sigma, Deisenhofen, Germany, P5899). Activation of HD11 was achieved by the addition of 5 μg LPS (Sigma, L-8275) per ml medium and subsequent cultivation over night [58]. Activated macrophages become postmitotic. Coverslips with attached cells were washed with PBS, fixed in 1,2% formaldehyde freshly made from paraformaldehyde [59] for 15 min, washed in PBS 3 × 3 min, incubated in 0,5% Triton X-100 in PBS for 20 min and equilibrated in 20% glycerol in PBS for 60 min. Dipping in liquid nitrogen and thawing at room temperature in 20% glycerol/PBS was performed five times. After washing 3 × 3 min in PBS and incubation in 0,1 M HCl for 10 min, coverslips were washed 2 × 3 min in 2 × SSC and stored in 50% formamid/ 2 × SSC for at least 1 h but usually overnight or longer at 4°C.

### FISH

Chromosome-specific paint probes for chicken chromosomes 1 and 8 were kindly provided by Dr. Felix Habermann and Dr. Johannes Wienberg, Munich. They were generated by flow sorting of metaphase chromosomes and subsequent degenerated oligonucleotide primed (DOP)-PCR [60]. They produce a uniform labeling of metaphase chromosomes. When the amplification products were repeatedly reamplified using the same PCR conditions, however, we noted after about a dozen rounds that the label on metaphase chromosomes became non-uniform in appearance indicating a reduction in complexity by the loss of sequences. The higher likelihood of retaining repetitive sequences was reflected by the finding that the by far brightest spot was found at the centromere as identified by the primary constriction in the DNA counterstain. To identify chromosome 1 centromeres together with completely delineated chromosome 1 territories, we used a mixture of an early amplification product with repeatedly reamplified probe. We thus obtained intense painting of the entire chromosome and a particular bright signal at the centromere (Figure 2a). In experiments where chromosomes 1 and 8 were cohybridized, detection of centromeres was not necessary and thus only early amplification products were used. DOP-PCR for amplification and labeling (biotin-16-dUTP for #1 and digoxigenin-16-dUTP for #8, both from Roche Applied Science, Mannheim, Germany) was performed as described [61]. The lysozyme gene domain is contained on the pPoly-III-i Lys-plasmid [62]. It was labeled by nick-translation with digoxigenin-dUTP. Chicken cot 1 DNA was prepared from liver using standard procedures. The same result was obtained for the chicken chromosome 8 paint probe (data not shown).

All DNA for a given assay was mixed, precipitated and solved in deionized formamide. The same volume of 20% dextransulfat in 2 × SSC was added. In experiments with *cLys *the following amounts of DNA were precipitated for each μl hybridization mix: 2 μl label-DOP-PCR product of an early amplification round of the #1 paint plus 2 μl of a highly amplified paint probe for centromere detection, 50 ng pPoly-III-i Lys, 2.5 μg cot 1 DNA. When paint probes for #1 and #8 were cohybridized, 2 μl label-DOP-PCR product of an early amplification round was used for each chromosome and supplemented with cot1 DNA as above. Denaturation was 5 min at 85°C. Preannealing with the cot 1 DNA was performed for 25 min at 37°C. For 3D FISH, coverslips with cells were denatured in 70% formamide for 3 min at 70°C, and placed immediately in ice-cold 50% formamide/2 × SSC. They were then incubated with 5 μl hybridization mix under a sealed 18 × 18 mm^2 ^coverslip at 37°C for 24 h-72 h. Air-drying was carefully avoided at all steps. Metaphase chromosomes were hybridized as described [37].

Detection was performed as described [63], using rabbit anti-digoxigenin (1:500) and goat anti-rabbit-Alexa488 (both from Sigma) and for biotin detection Avidin-Cy3 (Dianova, Hamburg, Germany). Slides were counterstained with DAPI and TOPRO-3 (Molecular Probes, Eugene, Oregon) and mounted in Vectashield (Vector Laboratories, Burlingame, CA).

### Confocal laser scanning microscopy

3D image stacks (8 bit) were recorded with a Leica TCS SP confocal laser scanning microscope equipped with an argon (488, 514 nm) and a HeNe laser (633 nm) (Leica Mikrosysteme, Bensheim, Germany). A 100 × N.A. 1,4 oil objective was used to obtain stacks with a voxel size of 0.08 × 0.08 × 0.24 μm. Nuclei with separated homologous chromosome territories were preferably selected for recording. To measure the chromatic aberration, 0.5 μm multi-color latex beads (Polysciences Europe, Eppelheim, Germany) were fed to activated macrophages. After phagocytosis the cells were fixed and embedded like 3D-FISH preparations. The beads were in the cytoplasma and thus their optical environment was closer to the situation of FISH signals than beads mounted directly on a glass cover slip. The chromatic aberration in x, y and z was corrected before the assignment of signals to categories or distance measurements were performed.

### Image analysis

The program used for object counting was first applied by Cremer et al. [44]. The original 8-bit gray level image stack is first subjected to Gaussian filtering and then normalized, i.e. the lowest existing gray value is set to zero, the highest to 255 and the values in-between are recomputed accordingly. A starting threshold of 20 was chosen and voxels above the threshold were determined. Of those, all touching voxels (26 voxel neighborhood) were combined to objects. Only structures with at least 10 voxels were regarded as 'objects' and included in the further analysis. After counting the objects and calculating the other parameters, the threshold was raised for 5 gray levels, object determination and calculation was repeated and so on until the highest applied threshold of 250 was reached. For statistical calculations, from each nucleus the maximum number of objects occurring at any threshold of 80 or higher was used. The restriction to thresholds of 80 or higher was made to confidently exclude background objects. Statistical inferences about the means of the maximum values of objects were based on one-way analyses of variance (ANOVA). Post-hoc comparisons generating p-values relied on Sidak tests. These test were performed with SPSS version 12 (SPSS Inc., Chicago, IL).

For the second parameter, for each nucleus at each threshold the ratio (object intensity)/(object surface voxels) was measured for all objects and averaged. Object surface voxels are defined as voxels belonging to an object and having at least one of the 26 neighbors not belonging to the object. The unit is 1/μm^2^. This value reflects the amount of intensity (chromatin) that is enclosed in a given surface area. Chromosome territories with a richly folded surface thus have a rather low value whereas compact, homogeneous territories have a higher value. Most nuclei had zero or few objects at very high threshold levels (Figure 5a,5b). Therefore, the calculation of meanvalues for the intensity/surface parameter was stopped when less than five nuclei with at least one object where left. For the third parameter, average amount of labeled chromatin per volume, the intensity of all voxels belonging to an object was summed up and divided by the number of object voxels and the average over the objects was computed.

Localization of *cLys *and centromere signals with regard to chromosome 1 territories: Image stacks were imported in ImageJ (freely available on the internet at [64]) and each fluorochrome was assigned to one channel of an RGB-Stack. A Gaussian Blur filter was applied before using the 'brightness & contrast' function to enhance signals and decrease background. The 'make montage' function was then used to show all planes of the RGB-stack side by side. For each gene or centromere signal the z-plane was selected where it was brightest. This plane was then used for the categorization (Figure 6a) [11]. The Mann-Whitney-U test from SPSS was used for statistical analysis. For the aneuploid cell line HD11 an evaluation was performed only if normal chromosomes were unequivocally distinguishable from the translocated p-arm (without centromere). The latter was excluded from further analysis.

High precision 3D-distance measurements [35,36]: Gravity centers of the signals were determined with Showpos, a program written by Kurt Sätzler, Heidelberg, for Silicon Graphics Workstations running under Irix. The 3D coordinates of *cLys *and the respective centromere were corrected for the chromatic aberration and the distance was calculated.

The quantitative assessment of 3D radial distributions of painted chromosome territories and the measurement of nuclear volumes was performed using a program developed by Dr. Johann von Hase, Heidelberg which is described in detail elsewhere [42]. To determine the statistical significance of radial distribution differences, we used the medians of each signal in each nucleus and applied the two-sided Kolmogorov-Smirnov test [65]. The same test was applied to nuclear volumes.

## Authors contributions

The study was designed by SD together with CB and TC. First experiments and development of techniques were performed by RM. S Stadler and VS performed 3D-FISH, microscopy, determination of cLys and centromere 1 localization and high precision distance measurements of all evaluated cells. RM carried out radial distribution statistics and volume measurements. All three were supervised by SD and TC. S Stein programmed and adapted the object counting program, supervised by CC. Object counting and statistical analysis was performed by SD. The manuscript was written by SD with substantial contributions by CB, TC and VS. All authors read and approved the final manuscript.

## Supplementary Material

Additional File 1 Radial distribution of chromosomes 1 and 8 in nuclei of different cell types. These graphs show the same curves as presented in Figure 7, but now all curves for chromosome 1 are combined in (a) and those for chromosome 8 are combined in (b). Click here for file
