# Supplementary figures and images for: The architecture of chicken chromosome territories changes during differentiation
Source: BMC Cell Biol. 2004 Nov 22;5:44. doi: 10.1186/1471-2121-5-44 (PMC535556; doi:10.1186/1471-2121-5-44)

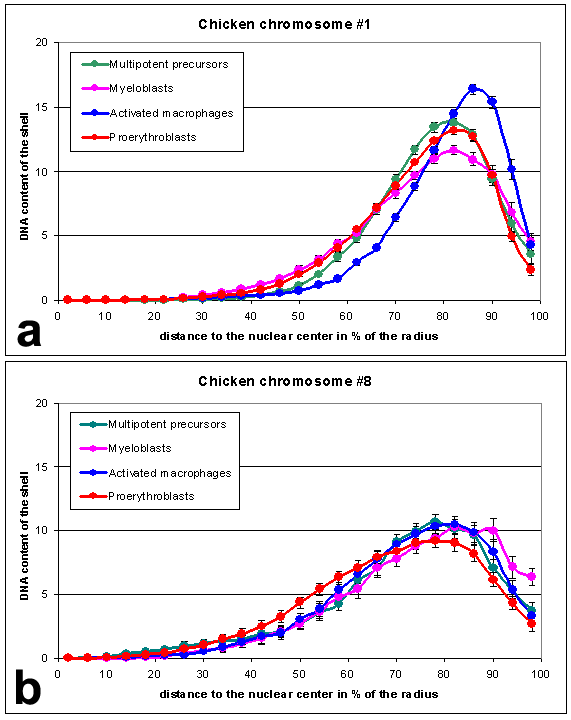

Supplement: Additional File 1 — Radial distribution of chromosomes 1 and 8 in nuclei of different cell types. These graphs show the same curves as presented in Figure 7, but now all curves for chromosome 1 are combined in (a) and those for chromosome 8 are combined in (b). [file 1471-2121-5-44-S1.tiff]
